# Lattice Thermal Conductivity in XMg_2_Sb_2_(X = Ca or Mg) Compounds: Temperature and High-Order Anharmonicity Effect

**DOI:** 10.3390/ma16237349

**Published:** 2023-11-25

**Authors:** Minghui Wu, Hongping Yang, Fengyan Xie, Li Huang

**Affiliations:** 1 College of Materials and Chemical Engineering, MinJiang University, Fuzhou 350108, Chinaxiefengyan@mju.edu.cn (F.X.); 2 Fujian Key Laboratory of Functional Marine Sensing Materials, Minjiang University, Fuzhou 350108, China; 3 Department of Physics, Southern University of Science and Technology, Shenzhen 518055, China; huangl@sustech.edu.cn

**Keywords:** lattice thermal conductivity, DFT, thermoelectric materials

## Abstract

The binary compound Mg_3_Sb_2_ (also written as MgMg_2_Sb_2_) exhibits a much lower lattice thermal conductivity ($\kappa_{L}$) than its ternary analog CaMg_2_Sb_2_, despite its relatively low mass density and simple crystalline structure. Here, we perform a comparative first-principles study of the lattice dynamics in MgMg_2_Sb_2_ and CaMg_2_Sb_2_ based on the density functional theory, together with the self-consistent phonon theory and the Boltzmann transport theory. We show that the modest anharmonicity of CaMg_2_Sb_2_ renders the three-phonon processes dominant, and the temperature dependence of *κ_L_* approximately follows the $T^{-1}$ relationship. In contrast, the strong quartic anharmonicity of MgMg_2_Sb_2_ leads to the ultralow *κ_L_* and weak temperature dependence, in agreement with the experimental observations. A comprehensive analysis reveals that the $\kappa_{L}$s in the two compounds are mainly carried by the acoustic phonons associated with the Sb atoms, and the different behaviors of $\kappa_{L}$ result from the chemical bond changes around Sb atoms, which bond more covalently with the Mg atoms than the Ca atoms and thus lead to high-order anharmonicity in MgMg_2_Sb_2_. These results give us insights into the understanding of the anomalous thermal transport in thermoelectric materials.

## 1. Introduction

Crystalline solids with inherently low thermal conductivity are technologically important for many fundamental applications, such as thermal barrier coatings, data storage devices, and especially thermoelectrics (TE) [1,2,3]. Indeed, almost all hitherto known thermoelectric materials with high energy conversion efficiency possess low lattice thermal conductivity ($k_{L}$). The $k_{L}$s in crystalline materials are generally explained by various phonon scattering mechanisms [4,5]. Usually, the physical origin of low $k_{L}$ is ascribed to the unconventional phonon-scattering processes induced by strong lattice anharmonicity or phonon–phonon couplings. Several rules of thumb, such as compounds with heavy elements (e.g., Bi_2_Te_3_ [6] and PbTe [7]), soft bonds (e.g., Tl_3_VSe_3_ [8], TlInTe_2_ [9]), and complex atomic structures (e.g., K_2_Bi_8_Se_13_ [10], Yb_14_MnSb_11_ [11], Ba_8_Ga_16_Ge_30_ [12], LaFeSb_12_ [13]), have been applied to screen materials with inherently low $k_{L}$s, since these features lead to low phonon velocities or high rates of phonon-phonon scattering [5]. A common practice for estimating the anharmonicity of solids is to obtain the Grüneisen parameter. The larger the Grüneisen parameter is, the stronger the anharmonicity, and so the lower the $k_{L}\;is$. Although the past decades have witnessed impressive progress in the understanding of thermal transport of crystalline materials, the underlying origin of the anomalously low $k_{L}$s present in some simple compounds still remains elusive.

The binary compound MgMg_2_Sb_2_, which crystallizes in the CaAl_2_Si_2_ structure type, has recently emerged as a promising TE material at intermediate temperatures [14,15,16,17,18]. Besides the excellent electronic transport properties, the unusually low $k_{L}$ in this simple crystalline phase also plays an equally important role in leading to the high TE performance. Despite its much lighter density than traditional TE compounds PbTe and Bi_2_Te_3_, MgMg_2_Sb_2_ exhibits a comparable $k_{L}$ at room temperature (1–1.5 W/mK). As one of the lightest compounds in the Zintl-type family, the surprisingly low $k_{L}$ of binary MgMg_2_Sb_2_ is anomalous compared with its isostructural ternary counterparts [19]. For example, the $k_{L}$ increases three times when replacing Mg at the octahedral site with heavier Ca or Yb [19], which is contrary to the common expectations that $k_{L}$ should be suppressed in compounds with more complex compositions and higher mass densities. It was also puzzling to note that the temperature dependence of $k_{L}$ in crystalline MgMg_2_Sb_2_ follows a relationship of *T*^−0.57^ [20], much weaker than the typical *T*^−1^ behavior above the Debye temperatures, which is usually expected if only taking the three phonon processes into consideration. Both the counterintuitive decrease and weak temperature dependence of $k_{L}$s in MgMg_2_Sb_2_ suggest that higher-order anharmonic interactions and temperature-dependent phonon frequency shifts should be included to understand the physics of the abnormal thermal conductivity in such simple and lightweight crystalline compounds.

In this work, we perform a comparative first-principles study of the lattice dynamics in MgMg_2_Sb_2_ and CaMg_2_Sb_2_ based on the density functional theory (DFT), together with the self-consistent phonon theory and the Boltzmann transport theory. We show that the modest anharmonicity of CaMg_2_Sb_2_ renders the three-phonon processes dominant, and the temperature dependence of $k_{L}$ approximately follows the $T^{-1}$ relationship. In contrast, the strong quartic anharmonicity of MgMg_2_Sb_2_ leads to an ultralow $k_{L}$ and a weak temperature dependence. In-depth analyses reveal that the $k_{L}$s in the two compounds are mainly carried by the acoustic phonons associated with the Sb atoms, and the origin of the unusual $k_{L}$ behaviors lies in the chemical bond changes of Sb atoms, which bond more covalently with the Mg atoms than the Ca atoms, and thus lead to strong high-order anharmonicity in MgMg_2_Sb_2_.

The paper is organized as follows: In Section 2, the details of our computational methods are presented. The lattice dynamics-related properties, such as elastic constants, phonon dispersions, lattice thermal conductivities, and chemical bond analysis are discussed in Section 3. Section 4 is the summary of our work.

## 2. Computational Methods

DFT calculations: All the first-principles calculations are performed using the projector augmented-wave (PAW) method as implemented in the Vienna ab initio Simulation Package (VASP) [21]. The exchange–correlation interaction is treated in the generalized gradient approximation (GGA) of Perdew, Burke, and Ernzerhof (PBE) [22]. The energy cutoff is set to 500 eV for the plane-wave basis sets. A Monkhorst-Packk-point mesh of 6 × 6 × 4 has been used to sample the Brillouin zone for the primitive cell, while only Γ point is used for the supercell calculations. The criterion for the relaxation is set to the minimum atomic force of less than 0.01 eV/Å. A 4 × 4 × 3 supercell containing 240 atoms is constructed to obtain the required force constants with a total energy convergence criterion of 10^−8^ eV. The maximally localized Wannier functions (MLWF) have been obtained using the Wannier90 Code [23,24] to understand the chemical bonding in the two compounds.

Molecular dynamics (MD) simulations: Ab initio MD simulations are conducted to investigate the lattice dynamics properties at finite temperatures. All the MD simulations are carried out with an NVT ensemble. Only the cases at temperatures of 400 K and 700 K have been investigated. The MD simulations last for at least for 30 ps with a time step of 2 fs, after the equilibration steps. The plane-wave energy cutoff and energy convergence criterion are set as 300 eV and 10^−4^ eV, respectively, to accelerate the calculations for the MD simulations.

Phonon and $\kappa_{L}$ calculations: Different methods have been used to calculate the required force constants. The second and third force constants at 0 K are calculated using the finite-displacement methods. We consider up to the seventh-nearest neighbors (7-NN) for the calculations of the third-order force constants, which leads to more than 700 configurations to be calculated. These configurations are constructed by displacing an atom from its equilibrium position by 0.01 Å. The second and third force constants can then be extracted by solving a least-square problem after obtaining the atomic forces from the DFT calculations for each configuration. At finite temperatures, we extract the force constants up to the fourth order by a state-of-the-art Compressing Sensor (CS) method. More information about this method can be found in reference [25]. We uniformly sampled 230 snapshots from the MD trajectory. In each sampled snapshot, we further displace all atoms by 0.1 Å in random directions to decrease the cross-correlations between the sampled configurations. All the harmonic and anharmonic force constants are obtained using the ALAMODE code [26].

Phonon dispersions are obtained via direct diagonalization of the dynamic matrix constructed from the second force constants and the $k_{L}$s are calculated using the Boltzmann transport equation (BTE) within the relaxation-time approximation

$$\kappa_{L}^{\mu \nu }\left(T\right)=\frac{1}{NV}\sum_{q}c_{q}\left(T\right)v_{q}^{u}\left(T\right)\tau_{q}\left(T\right)$$

where N is the number of atoms and *V* is the volume of the unit cell, $c_{q}\left(T\right)$ is the mode-specific heat, $v_{q}\left(T\right)$ the group velocity, and $\tau_{q}\left(T\right)$ the lifetime of phonon *q*. The phonon lifetime *τ* is evaluated from the imaginary part of the bubble self-energy, which only takes the third force constants into consideration. At finite temperatures, the renormalization of the vibrational frequency by the quartic anharmonicity is considered using the self-consistent phonon (SCP) method implemented in the ANPHON code [27]. The phonon dispersions and the corresponding quartic anharmonicity corrected $k_{L}$s at different temperatures are then obtained using the corresponding force constants at finite temperatures. For all our $k_{L}\;$thermal conductivity calculations, the *q*-mesh is set to 30 × 30 × 20, which is dense enough to obtain the convergent result.

## 3. Results and Discussion

### 3.1. Crystal-Structure and Elastic Properties

**Figure 1:**
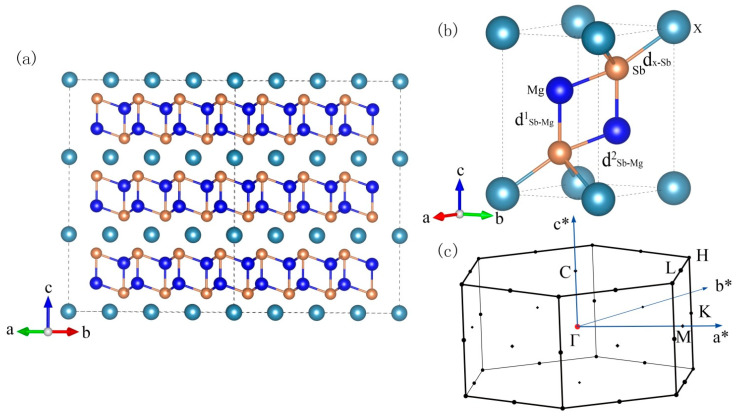
(**a**) Supercell (4 × 4 × 3 of the unit cell) of XMg_2_Sb_2_ (X = Mg^1^, Ca) compounds. Please note that we added a superscript to the Mg atom at the X position. (**b**) The primitive cell and (**c**) the symmetric point of the first Brillion Zone.

As shown in Figure 1, the layered CaAl_2_Si_2_-type compounds XMg_2_Sb_2_ (X = Mg^1^ or Ca) belong to the Zintl family with a trigonal lattice of the $D_{3d}^{3}$($P\bar{3}m1$) symmorphic space group. The structure is commonly characterized by the alternating covalently bounded anionic [Mg_2_Sb_2_]^2−^ layers and loosely bounded two-dimensional cationic X^2+^ (Ca or Mg^1^) ion layers. In the following, we refer to Mg at the highly distorted octahedral X site as Mg^1^. The Mg-Sb bond lengths differ significantly between the two sites. Table 1 collects the equilibrium lattice constants of XMg_2_Sb_2_ compounds, which are all in good agreement with the experimental values. It can be seen that the average atomic volume and the ratio c/a is larger in CaMg_2_Sb_2_ than in MgMg_2_Sb_2_, which indicates that CaMg_2_Sb_2_ has a more loosely packed structure than MgMg_2_Sb_2_. A larger average atomic volume generally can afford more dynamic freedom for the atomic movement, and so has a greater tendency to induce lattice dynamic abnormality. Also, considering the higher mass density and more complex compositions of the ternary compound CaMg_2_Sb_2_ than the binary MgMg_2_Sb_2_, one may expect that the former should have a lower $k_{L}$ than the latter. However, contrary to common intuitions, recent experiments observed that the $k_{L}$ of MgMg_2_Sb_2_ is three-times lower than that of CaMg_2_Sb_2_ [14].

The elastic properties of solids are the macroscopic reflection of the intrinsic atomic lattice dynamics and can be used to evaluate the $\kappa_{L}$ [30]. Here, using the first-principle-based perturbation theory, the elastic constants of these compounds are calculated and listed in Table 1, and then, the Hill average of the bulk (B), shear (G), and Young moduli (E) are further evaluated [31]. As shown in Table 1, unlike the typical layered thermoelectric materials, such as SnSe and Bi_2_Te_3_, the anisotropy of the elastic properties of XMg_2_Sb_2_ compounds are quite small (C_11_/C_33_ ≈ 1.2 for CaMg_2_Sb_2_ and 0.9 for MgMg_2_Sb_2_), indicating that the two compounds actually are more akin to the bulk materials than the layered materials. We note that the calculated elastic constants of Mg_3_Sb_2_ are a little bit smaller than that of CaMg_2_Sb_2_, consistent with the situation of $k_{L}$.

Thermal conductivity ingredients, such as the longitudinal (*v*_L_) and transversal (*v*_T_) sound velocity, and Debye temperature (Θ_D_) can be further derived using the following equations:

$$\rho v_{L}^{2}=B+\frac{4}{3}G,\;\rho v_{T}^{2}=G,\;\Theta_{D}=\frac{\hbar v_{A}{\left(6\pi^{2}n\right)}^{1/3}}{k_{B}}$$

where *ρ* is the density, *n* is the atomic number per volume, and *v*_A_ is the average sound velocity ($\frac{3}{v_{A}^{3}}=\frac{2}{v_{L}^{3}}+\frac{1}{v_{T}^{3}}$). These results are also presented in Table 1. After comparing these parameters with other low-$\kappa_{L}$ compounds, such as PbX(X = Se, Te) [32], BiCuXO(X = S, Se and Te) [33], SnSe [34], Cu_2_Se [35], TlXTe_2_(X = Ga, In) [9], and Tl_3_VSe_4_ [8], one can find that these values for XMg_2_Sb_2_ compounds are at a low level, hinting that the $\kappa_{L}$ of XMg_2_Sb_2_ (X = Mg^1^ or Ca) compounds is small, especially for MgMg_2_Sb_2_.

### 3.2. Phonon Dispersion

**Figure 2:**
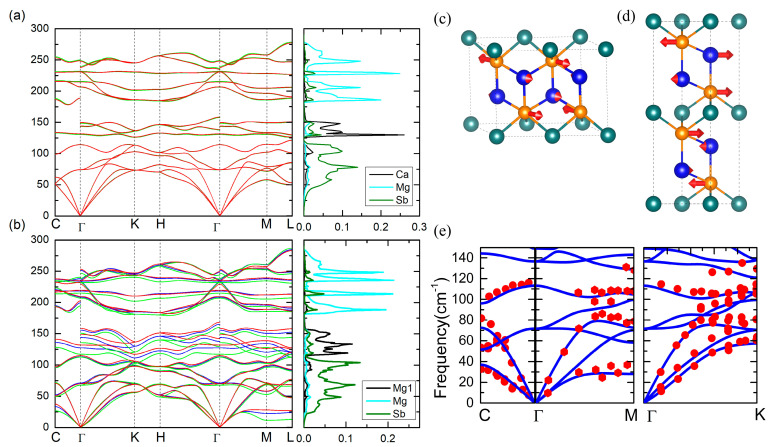
Phonon dispersions and atomic project density of phonon state (pod’s) of CaMg_2_Sb_2_ (**a**) and MgMg_2_Sb_2_ (**b**). For phonon dispersion, the green line denotes the result of 0 K, the blue line 400 K, and the red line 700 K. The pDOS are obtained from the phonon dispersion of the result at 0 K. Please note that the color of the pDos line is not related to the temperature. (**c**,**d**) Vibration modes of the lowest acoustic mode at M and C points. In (**e**), the blue line is the phonon dispersion at 300 K obtained by the self-consistent phonon method and the red points are the experimental results obtained from ref. [36].

The calculated phonon dispersions at different temperatures along the high-symmetry lines in the first Brillion Zone (BZ) are plotted in Figure 2. The primitive cell of XMg_2_Sb_2_ consists of one formula (five atoms) having a total of 15 phonon branches, among which 3 are acoustic and 12 are optical modes. At the high-symmetry Γ point, the factor group analysis yields

$$\begin{array}{l} D_{3d}^{}=E_{u}⊕A_{2u}\;\left(\mathrm{acoustic}\right)\\ ⊕2E_{g}⊕2A_{1g}⊕2E_{u}⊕A_{2u}⊕A_{2u}\;\left(\mathrm{optic}\right). \end{array}$$

Except for the three acoustic modes, the optical modes can be further classified into two categories: Raman active (R) modes and inferred (IR) active modes. In Table 2, we present the calculated results for different temperatures with a detailed description of the eigen displacements and participating atoms for each mode in order to facilitate the comparison with experimental results. Based on the eigen displacement information, one can qualitatively evaluate the effect of either the point defects or doping to these modes. We find that the defects or doping at X sites would strongly affect the IR active modes, while the effect of defects or doping related to the [Mg_2_Sb_2_]^2−^ layer can be experimentally observed in the Raman Spectra. The influence of temperature on these R or IR active modes of these two materials is different. As can be seen in Table 2, most of these modes are blueshift as temperature increases, and much larger changes with temperature can be observed in MgMg_2_Sb_2_ compared to CaMg_2_Sb_2_, indicating a stronger anharmonicity of MgMg_2_Sb_2_.

We note that there exists splitting between the longitudinal and transverse optical modes (LO-TO splitting) at the Γ point in the two compounds. The magnitude of the LO-TO splitting depends on the Born effective charge (BEC) and the dielectric screening function of the Coulomb interaction determined by the electronic part of the dielectric constants. The BEC tensor, described as the static and dynamic polarization, is diagonal and reduced into two independent values, $Z_{aa}^{*}=Z_{bb}^{*}=Z_{X⊥}^{*}$ and $Z_{cc}^{*}=Z_{X∥}^{*}$, which are listed in Table 3, along with the nominal charges obtained by the Bader analysis method for each element in the two compounds. One can see that the overall chemical bonding in MgMg_2_Sb_2_ is less ionic than that in CaMg_2_Sb_2_. Particularly, the electronic dynamics polarization effect on Sb atoms in MgMg_2_Sb_2_ is a little larger than that in CaMg_2_Sb_2_, especially along the in-plane direction, indicating covalent chemical bonds around Sb atoms in MgMg_2_Sb_2_ are a little bit stronger. The obtained BEC and dielectric constants show small differences along the c and a/b axis indicating that these two compound are fairly isotropic, in accordance with the elastic properties. We also note that the lattice contribution to the dielectric response (ε^0^–ε^∞^) in MgMg_2_Sb_2_ is almost twice as large as that in CaMg_2_Sb_2_, again indicating the presence of lower-frequency phonon modes in MgMg_2_Sb_2_ compared to CaMg_2_Sb_2_.

The phonon dispersions at different temperatures (T = 0, 400, 700 K) and the atom-projected phonon density of states (*p*DOS) at 400 K for the two compounds are shown in Figure 2. It can be seen that the contributions from different atoms are spectrally well separated. For both compounds, the vibrations of Mg atoms in the [Mg_2_Sb_2_]^2−^ layer mainly occupy the high optical frequency range and decouple from other atoms with a large gap of more than 50 cm^−1^, Mg^1^ or Ca contributes the intermediate frequency modes, and Sb dominates the lowest frequency region, indicating that the Sb atoms play a critical role in the thermal conductivity. One can also note that there exist significant differences between the phonon spectra of these two compounds: (i) compared with CaMg_2_Sb_2_, the acoustic phonon modes in MgMg_2_Sb_2_, which are mainly contributed by Sb atoms, become softer at the Brillouin zone boundary M-, L-, and C-point; and (ii) the *p*DOS of Mg^1^ contributes more to the lower energy side and is substantially broader than that of Ca, and a small gap of 10 cm^−1^ appearing between the *p*DOS of Ca and Sb in CaMg_2_Sb_2_ phonon spectra disappears in MgMg_2_Sb_2_. These differences suggest that the interactions between Mg^1^-Sb are much weaker than Ca-Sb, which was also reported in earlier studies [37].

A further scrutinization of the temperature response of the phonon dispersions in the two compounds also reveals different behavior. As can be seen in Figure 2b, most phonon modes of MgMg_2_Sb_2_ generally stiffen with increasing temperature. Specifically, the transverse acoustic phonons become significantly stiffer upon warming, especially at the Brillouin zone boundary C-, M-, and L-point. However, the phonon spectra of CaMg_2_Sb_2_ only show negligible changes with temperature, as displayed in Figure 2a. The sound velocities derived from the slopes of the acoustic branches of the phonon dispersions at the Γ point are also listed in Table 3, which are consistent with the those obtained by the elastic parameters (Table 1). It is thus not surprising to see that the sound velocities of MgMg_2_Sb_2_ are smaller than the counterparts of CaMg_2_Sb_2_. In Figure 2e, we compare the phonon dispersions of MgMg_2_Sb_2_ at 300 K obtained by the self-consistent phonon method and the corresponding experimental results from the inelastic X-ray scattering (IXS) [36]. A very good agreement can be observed between the calculated and experimental results, and the small deviation might be due to other factors such as lattice expansions and defects inherent in the experimental samples, which are not considered here.

### 3.3. Lattice Thermal Conductivity

**Figure 3:**
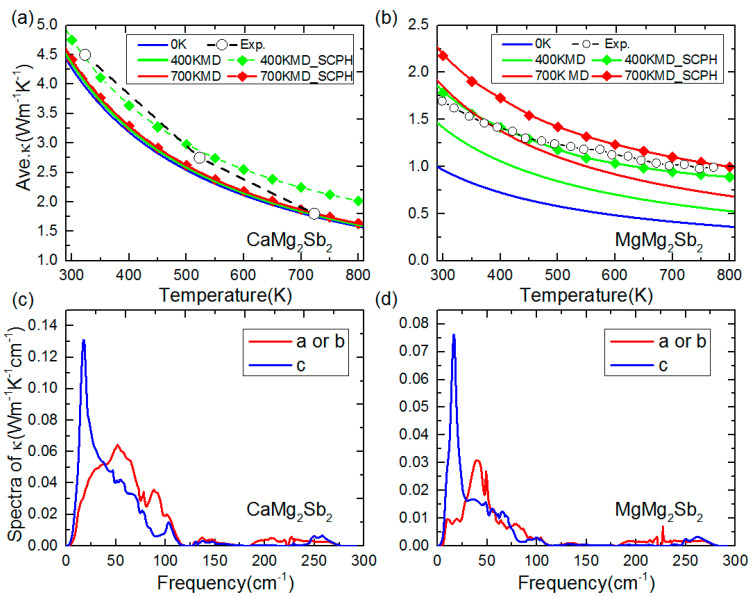
Thermal conductivity vs. temperature for CaMg_2_Sb_2_ (**a**) and MgMg_2_Sb_2_ (**b**). The experimental results are obtained from reference [20,28]. Spectra of thermal conductivity at 400 K temperature for CaMg_2_Sb_2_ (**c**) and MgMg_2_Sb_2_ (**d**). The 700 K MD results for spectra shown in Figure S4.

We now move on to investigate the physics behind the anomalously low $\kappa_{L}$ of MgMg_2_Sb_2_, compared with that of CaMg_2_Sb_2_. Figure 3 plots the calculated $\kappa_{L}$s for the two compounds in the temperature range of 300 to 800 K. The force constants (FCs) used in the calculations are obtained at three different temperatures (0 K, 400 K, and 700 K), and the lattice dynamical effects of the fourth-order anharmonicity have also been considered. As seen in Figure 3a, the predicted $\kappa_{L}$s for CaMg_2_Sb_2_ using 0 K force constants (harmonic description of the phonon energy and lifetime considering only three-phonon interactions) are quite close to the experimental data, and the temperature dependence of $\kappa_{L}$ largely follows the typical *T*^−1^ relationship, indicating that the dominant phonon scattering mechanism in this compound is the three-phonon scattering process. The calculated $\kappa_{L}$s using the FCs extracted from AIMD at 400 K and 700 K, respectively, are almost the same as those obtained using the FCs at 0 K. To probe the anharmonic renormalization arising from high-order (or particularly quartic) anharmonicity in this compound, the $\kappa_{L}$s are also obtained based on the self-consistent phonon (SCPH) theory including both three- and fourth-phonon interactions. The resulting $\kappa_{L}$s show an even better agreement with the experimentally measured values. As for MgMg_2_Sb_2_, we note that calculations using the FCs at 0 K seriously underestimate the $\kappa_{L}$s [see Figure 3b]. For example, the predicted $\kappa_{L}$ at 300 K is 1.0 Wm^−1^K^−1^, much less than the experimental value of 1.8 Wm^−1^K^−1^. Similar phenomena have also been reported in other simple crystalline compounds, such as Tl_3_VSe_4_ [8] and TlGaTe_2_ [9]. The calculated $\kappa_{L}$s can be enhanced using the FCs extracted from AIMD at 400 K, but still much smaller than the experimentally measured ones. A significantly improved agreement with the experiments in both the magnitude and temperature dependence of $\kappa_{L}$ can be achieved by further including fourth-phonon processes [see 400 KMD_SCPH line in Figure 3b], indicating that the quartic anharmonicity should be taken into consideration to account for the unusual $\kappa_{L}$ of MgMg_2_Sb_2_. Again, we note that the calculated $\kappa_{L}$ shows a very small anisotropy for the two compounds considered here, with the out-of-plane $\kappa_{L}$ (along the c axis direction) marginally larger than the in-plane one (along the a or b axis direction), as shown in Figures S1 and S2 in the Supplemental Materials.

The spectra $\kappa_{L}$s of the two compounds at 400 K and 700 K are displayed in Figure 3c,d and Figure S4, respectively. It can be seen that the overall $\kappa_{L}$s are dominated by the phonons with frequencies of less than 100 cm^−1^ in both compounds. As seen from the calculated *p*DOS shown in Figure 2, the low-frequency phonons are mainly contributed to by Sb atoms in the two compounds. Furthermore, we note that, despite an almost isotropic $\kappa_{L}$ present in both compounds, the distribution of the spectrally decomposed $\kappa_{L}\left(\omega \right)$ along the *c* axis is quite different from that in the *ab* plane. The $\kappa_{L}\left(\omega \right)$ along the *c* axis shows a sharp peak at the low frequency side, corresponding to the lowest transverse acoustic branches at the M- and C-point (see Figure 2a,b). The corresponding vibration modes at the two points are illustrated in Figure 2c and Figure 2d, respectively, which clearly show that the displacement of Sb atoms dominates the soft vibrational modes at these q points.

**Figure 4:**
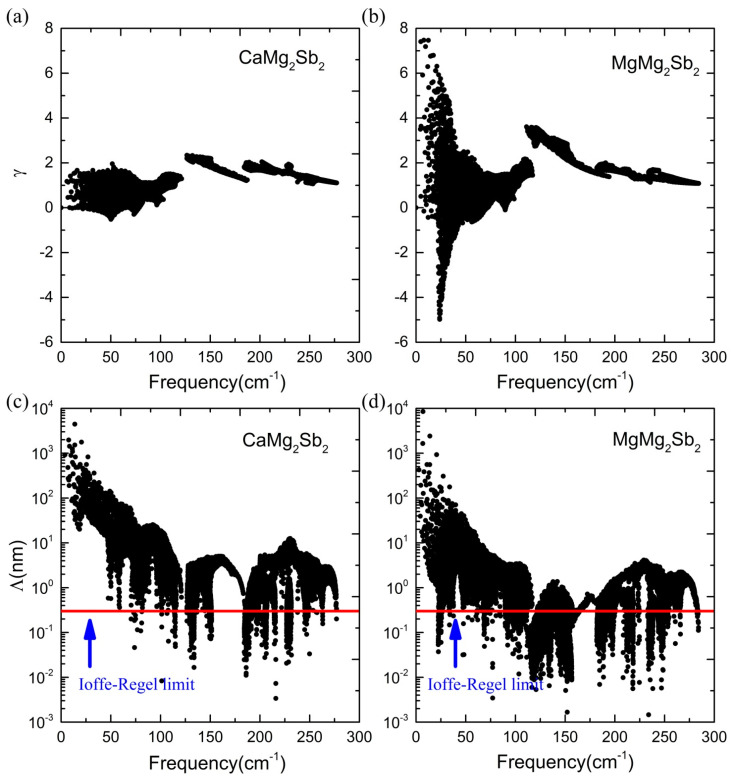
Grüneisen parameters and mean free path vs. frequency obtained from the data calculated using the 400 K ab initio MD calculated force constants. The red line denotes the Ioffe–Regel limit. The 700 K MD results are shown in Figure S5. (**a**) Grüneisen parameters for CaMg2Sb2, (**b**) Grüneisen parameters MgMg2Sb2, (**c**) mean free path for CaMg2Sb2, and (**d**) mean free path for MgMg2Sb2.

To better understand the unusually low $\kappa_{L}$ of MgMg_2_Sb_2_, especially compared with the isostructural CaMg_2_Sb_2_, we further calculate the mode Grüneisen parameters ($\gamma_{q}$) in the two compounds, which are defined as $\gamma_{q}=-\partial \ln\omega_{q}/\partial \ln V$ and commonly used to characterize the extent of anharmonicity. Here, we use the FCs from the AIMD at 400 K and 700 K to calculate all $\gamma_{q}$s, respectively. The resulting $\gamma_{q}$s as a function of frequency at 400 K are shown in Figure 4a for the two compounds. It can be seen that MgMg_2_Sb_2_ has the largest absolute values of $\gamma_{q}$ in the low-frequency acoustic regime (≤75 cm^−1^), and the corresponding phonon modes contribute strongly to phonon–phonon scattering. In contrast, the large values of $\gamma_{q}$ for CaMg_2_Sb_2_ appear in the optical frequency range from 125 to 150 cm^−1^. The overall $\gamma_{q}$s have a much larger absolute value in MgMg_2_Sb_2_ than in CaMg_2_Sb_2_ in the low- and medium-frequency range, while the values in the high-frequency region are close to each other. The similar features of $\gamma_{q}\left(\omega \right)$ are also observed at 700 K, as displayed in Figure S5a,b. Considering that the phonon modes in the low- and medium-frequency regime are almost exclusively contributed by Sb and Mg^1^ or Ca atoms (see Figure 2a), one can infer that the anharmonicity of the Mg^1^-Sb interaction in MgMg_2_Sb_2_ is much stronger than that of Ca-Sb in CaMg_2_Sb_2_, which thus significantly suppress the $\kappa_{L}$ of MgMg_2_Sb_2_ relative to that of CaMg_2_Sb_2_, overcoming the expected mass trends between Mg and heavier Ca.

**Figure 5:**
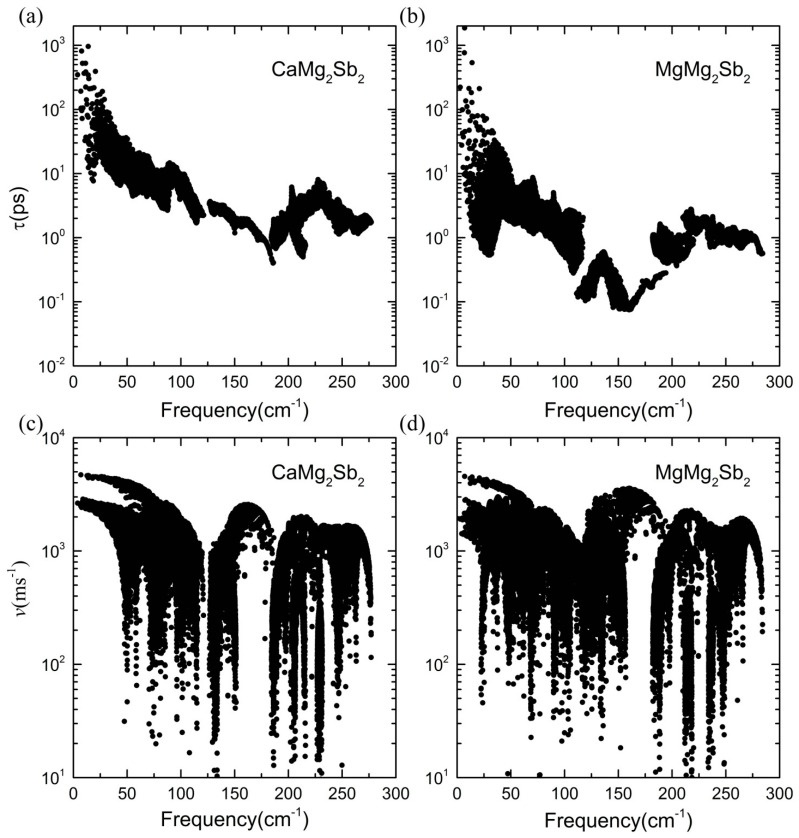
Phonon lifetime and group velocity vs. frequency obtained from the data calculated using the 400 K ab initio MD calculated force constants. The 700 K MD results are shown in Figure S6. (**a**) lifetime for CaMg2Sb2, (**b**) lifetime MgMg2Sb2, (**c**) group velocity for CaMg2Sb2, and (**d**) group velocity for MgMg2Sb2.

The distributions of the mean free path (MFP, Λ) at 400 K and 700 K are shown in Figure 4c,d and Figure S5c,d, respectively. It can be seen that the low-frequency phonons have the longest MFPs at both temperatures in the two compounds, and the overall MFP in MgMg_2_Sb_2_ is relatively shorter than that in CaMg_2_Sb_2_, but it is still much larger than the so-called Ioffe–Regel limit in the low-frequency regime (≤100 cm^−1^). Since the MFP depends on the combined effects of phonon velocity and lifetime, we also compare the phonon velocity and lifetime of the two compounds at 400 K and 700 K in Figure 5 and Figure S6, respectively. It can be found that the phonon group velocities of MgMg_2_Sb_2_ are very close to the counterparts of CaMg_2_Sb_2_ in the whole frequency range, while the overall phonon lifetime in MgMg_2_Sb_2_ is smaller than that in CaMg_2_Sb_2_.

### 3.4. Chemical Bonding

The above analyses show that $\kappa_{L}$s of XMg_2_Sb_2_ compounds are mainly contributed by the low-frequency phonon modes, which are dominated by the vibrations of Sb atoms. It is thus unexpected that replacing X-site Mg^1^ with Ca can dramatically affect the phonon transport and yield a threefold increase in the $\kappa_{L}$ in CaMg_2_Sb_2_. To provide further insight into the anomalous behavior of $\kappa_{L}$ in XMg_2_Sb_2_, we first explore the direct bonding character in the two compounds. As shown in Figure 1, the three nonequivalent atoms X, Mg, and Sb have quite different coordination environments: the X-site is coordinated by six equivalent Sb atoms, and Mg in the anionic [Mg_2_Sb_2_]^2−^ layer is coordinated by four Sb atoms forming vertical bonds (parallel to the c axis) $d_{\mathrm{Mg}\text{-}\mathrm{Sb}}^{1}$ and tilt bonds $d_{\mathrm{Mg}\text{-}\mathrm{Sb}}^{2}$. So intuitively, it may be thought that the anionic [Mg_2_Sb_2_]^2−^ layer and the cationic X ions layer are bridged by the Sb atoms. Detailed information on these bonds is presented in Table 1. It can be found that the bond lengths of $d_{\mathrm{Mg}\text{-}\mathrm{Sb}}^{1}$ in the two compounds are almost the same despite the fact sthat the cell volume of MgMg_2_Sb_2_ has contracted about 9.6% in comparison with CaMg_2_Sb_2_, implying that the chemical bond difference of these two compounds mainly comes from $d_{X\text{-}\mathrm{Sb}}^{}$ and $d_{\mathrm{Mg}\text{-}\mathrm{Sb}}^{2}$. To further understand the nature of the chemical bond formed in these two compounds, we turn to the maximally localized Wannier functions (MLWFs).

**Figure 6:**
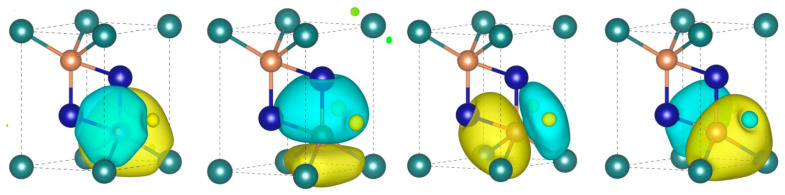
The MLWFs of one Sb atom in MgMg_2_Sb_2_ compound. We only show one of the Sb atoms since the MLWFs for the other Sb atom are the same due to symmetry. As the MLWFs contour for both of our considered compounds are alike, we only show the case of MgMg_2_Sb_2_. However, the reader can find all of the MLWFs in Figure S7. We also show the distribution of the MLWF center in Figure S8.

The obtained MLWFs around one Sb atom are plotted in Figure 6, and the other MLWFs can be found in Figure S7. The ingredients, MLWF centers (WFC) offset distance (*d*_WFC-X_ et al.), and spreads (Ω) featured in the MLWFs are listed in Table 1. All of the MLWFs show *s-p* electron hybridization, consistent with the valence configuration of Ca, Mg, and Sb species. For both of our considered compounds, the WFCs are located quite nearby the atom site (See Figure S8), indicating that the chemical bonding of these two compounds is almost ionic, consistent with the more sophisticated topological electron density analysis [38]. However, a subtle difference still exists for these two compounds. Compared with the Sb atoms, the MLWFs of X and Mg atoms are more localized indicating that the iconicity of these atoms is stronger. The MLWFs around the Sb atoms definitely indicate that the Sb atoms bridge the X and Mg atoms. For the X ions layer, compared with MgMg_2_Sb_2_, $d_{\mathrm{WFC}\text{-}X}$ and $\Omega_{X}$ of the Ca atoms are much larger than for the Mg^1^ atom, since the Ca atoms have a large radius. For the [Mg_2_Sb_2_]^2−^ layer, MLWFs ($d_{\mathrm{WFC}\text{-}X}$ and $\Omega_{X}$, Table 1) of Mg atoms do not show any difference in these two compounds, while the difference of these two compounds mainly originates from the Sb atoms. The WFC-atom distance and spread of MLWF for the Sb atom (Table 1) show that the Sb atom in MgMg_2_Sb_2_ is a little more covalent than Sb in the CaMg_2_Sb_2_ compound. This assertion is in agreement with the discussion of the Born effective charge and Bader charge.

## 4. Summary and Conclusions

To summarize, the lattice dynamics-related properties of the layer-like zintl compounds XMg_2_Sb_2_ (X = Mg^1^ or Ca) have been investigated using the first-principle-based phonon self-consistent and Boltzmann transport theory method. Contrary to intuition, the anisotropy of lattice dynamics-related properties of these two compounds turns out to be very small, suggesting that XMg_2_Sb_2_ (X = Mg^1^ or Ca) are intrinsically more bulk-like materials. The anharmonicity of MgMg_2_Sb_2_ is much larger than that of CaMg_2_Sb_2_. The modest anharmonicity of CaMg_2_Sb_2_ renders the three-phonon process dominant, leading to its intrinsic lattice thermal conductivity ($\kappa_{L}$) of approximately *T*^−1^ trend and faint temperature dependence. The strong anharmonicity of MgMg_2_Sb_2_ results in fierce temperature dependence of its atomic interaction and the significance of the high-order (fourth order) phonon process to accurately predicate its $\kappa_{L}$. The mollification of MgMg_2_Sb_2_ $\kappa_{L}$ form *T*^−1^ trend is caused by a combination effect of temperature and high-order anharmonicity. The $\kappa_{L}$ for both of our compounds are mainly carried by low-frequency acoustic phonon modes, dominantly occupied by the vibration of Sb atoms. The chemical bond analysis shows that, contrary to the other atoms, the chemical bonds around Sb atoms are a little bit more covalent. We conclude that although the vibration of X atoms does not directly contribute to the $\kappa_{L}$, its electronegativity can tune the ionicity (covalency) of the chemical bond around the Sb atoms. Compared with Ca, Mg disturbs the bonds around Sb atoms by adding a little more covalency to it, leading to MgMg_2_Sb_2_ having more anharmonicity.

## Figures and Tables

**Table 1:** Lattice parameters, direct chemical bond distance ($d_{X\text{-}\mathrm{Sb}}^{}$, $d_{\mathrm{Mg}\text{-}\mathrm{Sb}}^{1}$ and $d_{\mathrm{Mg}\text{-}\mathrm{Sb}}^{2}$ in Å), Wannier Function center (WFC) offset distance (d_WFC-Sb_, d_WFC-Mg_, and d_WFC-X_ in Å) and Spread (Ω, Å^2^), elastic constants Cij, bulk (B), shear (G), and Yang (E) moduli (in GPa), elastic constants derived longitudinal, transversal, and average sound velocity (v_l_ v_t_ $\bar{v}$ in m/s) as well as Debye temperature (ΘD in K) for XMg_2_Sb_2_ (X = Mg1, Ca) compounds.

Compound	Lattice Constant			Direct Chemical Bond					MLWF							
	A (Å)	c/a		$d_{X\text{-}\mathrm{Sb}}^{}$	$d_{\mathrm{Mg}\text{-}\mathrm{Sb}}^{1}$		$d_{\mathrm{Mg}\text{-}\mathrm{Sb}}^{2}$		d_WFC-Sb_		d_WFC-Mg_		d_WFC-X_	Ω_Mg_	Ω_X_	Ω_Sb_
CaMg_2_Sb_2_	4.69 4.65 ^a^	1.62 1.63 ^a^		3.28	2.94		2.87		0.75, 0.82 *		0.18		0.32	0.14	0.43	2.91, 3.34 *
MgMg_2_Sb_2_	4.60 4.58 ^b^	1.58 1.58 ^b^		3.11	2.93		2.82		0.72, 0.88 *		0.18		0.18	0.14	0.14	3.15, 3.50 *
Elastic properties																
	C_11_	C_12_	C_13_	C_33_	C_44_	C_23_		C_55_	B	G		E	$v_{L}$	$v_{T}$	$\bar{v}$	Θ_D_
CaMg_2_Sb_2_	77.9	27.0	20.9	66.1	25.4	20.9		26.7	40.0	26.0		64.0	4423 4330 ^a^	2610 2716 ^a^	3536	34.1
MgMg_2_Sb_2_	71.0	37.0	21.7	76.0	17.0	21.7		14.0	42.1	18.2		47.7	4099	2147	2960	28.8

^a^ Ref. [28], ^b^ ref. [29]. * denote the corresponding MLWF stretched along the c direction.

**Table 2:** The calculated optical phonon frequency at the high-symmetry point Γ along with the mode symmetries, characters, and description of involved atoms in the eigen displacements (Figure S3 in Supplemental Materials) at different temperatures. The direction of atom vibrations a, b and c refer to Figure 1a. Δω = ω (T, SCPH) − ω (T). ω (T, SCPH) means the phonon frequency calculated using phonon self-consistent method.

Mode	T(K)	CaMg_2_Sb_2_			MgMg_2_Sb_2_		
		ϖ (cm^−1^)		Involved Atoms: Direction	ϖ (cm^−1^)		Involved Atoms: Direction
		DFT	Δω		DFT	Δω	
E_g_ (R, 1)	0	74.0	-	Mg, mainly Sb: One Mg, Sb pair a (b), One Mg, Sb pair minus a (b)	67.6	-	Mg, mainly Sb: One Mg, Sb pair a (b), One Mg, Sb pair minus a (b)
	300	74.1	0.4		71.2	0.2	
	700	74.6	0.2		69.8	1.9	
A1g (R)	0	114.3	-	Sb: One Sb c, One Sb minus c	112.3	-	Sb: One Sb c, One Sb minus c
	300	114.2	1.1		112.9	1.1	
	700	114.1	2.2		114.6	1.3	
E_u_ (IR, TO)	0	130.6	-	mainly Ca, Sb: Ca a, Sb minus a	116.9	-	mainly Mg^1^, Sb:Mg^1^a, Sb minus a
	300	131.5	4.1		127.1	9.4	
	700	132.4	7.8		131.6	14.6	
E_u_ (IR, LO)	0	143.4	-	mainly Ca, Sb: Ca b, Sb minus b	150.6	-	mainly Mg^1^, Sb:Mg^1^ b, Sb minus b
	300	143.9	3.9		155.5	6.9	
	700	144.5	7.0		158.2	11.1	
A2u (IR, 1)	0	148.3	-	All, Ca(most): Ca c, Sb minus c, Mg c	135.5	-	All, Mg^1^ (most): Mg^1^ c, Sb minus c, Mg c
	300	149.5	3.8		143.6	6.7	
	700	151.4	6.5		148.7	9.1	
A1g (R)	0	206.6	-	Mg: One Mg c, One Mg minus c	206.7	-	Mg: One Mg c, One Mg minus c
	300	206.8	3.5		208.3	2.2	
	700	206.5	6.4		209.3	5.3	
Eu (IR, TO)	0	215.6	-	Sb, Mg(most): Mg a, Sb minus a	214.8	-	Sb, Mg(most): Mg a, Sb minus a
	300	214.5	2.2		218.1	−0.8	
	700	217.3	3.0		219.4	−0.2	
Eu (IR, LO)	0	231.6	-	Sb, Mg(most): Mg b, Sb minus b	231.8	-	Sb, Mg(most): Mg b, Sb minus b
	300	230.9	3.0		233.9	1.5	
	700	234.3	4.3		234.8	2.4	
E_g_ (R, TO)	0	231.6	-	Sb, Mg(most): one Mg and Sb pair a, One Mg and Sb pair minus a,	231.8	-	Sb, Mg(most): one Mg and Sb pair a, One Mg and Sb pair minus a
	300	230.9	3.0		233.9	1.5	
	700	234.3	4.3		234.8	2.4	
E_g_ (R, LO)	0	248.6	-	Sb, Mg(most): one Mg and Sb pair b, One Mg and Sb pair minus b	246.5	-	Sb, Mg(most): one Mg and Sb pair b, One Mg and Sb pair minus b
	300	247.8	1.9		251.3	−0.1	
	700	250.0	3.1		253.0	0.7	
A_2u_ (IR)	0	239.5	-	Ca, Mg: Ca c, Mg minus c	239.2	-	Mg^1^, Mg: Mg^1^ c, Mg minus c
	300	237.8	2.7		236.7	1.2	
	700	238.5	4.5		230.8	3.4	

**Table 3:** Calculated Born effective (Z*), ionic charge $Z_{B}^{*}$ (obtained by Bader method), optical (ε^∞^) and static (ε^0^) dielectric constants, and longitudinal, transversal (*v_l_*, *v_t_* in m/s), and average sound velocity $\left[\left(v_{a}+v_{b}+v_{c}\right)/3\right]$ obtained from the phonon dispersion at room temperature for XMg_2_Sb_2_ (X = Mg^1^ or Ca) compounds.

Compounds	$(Z_{X⊥}^{*}$$,\;Z_{X∥}^{*}$)			$Z_{B}^{*}$			$\left(\varepsilon_{⊥}^{\infty },\varepsilon_{∥}^{\infty }\right)$	$\left(\varepsilon_{⊥}^{0},\varepsilon_{∥}^{0}\right)$	$\left(v_{l},v_{t}\right)$(m/s)		
	X	Mg	Sb	X	Mg	Sb			a/b	c	Ave.
CaMg_2_Sb_2_	(2.52, 2.75)	(1.87, 1.74)	(−3.14, −3.11)	1.38	2.00	−2.69	(6.56, 8.03)	(11.0, 11.0)	(2575, 4657)	(2644, 4749)	(2598, 4687)
MgMg_2_Sb_2_	(3.34, 3.20)	(1.91, 1.84)	(−3.58, −3.44)	2.00	2.00	−3.00	(4.97, 6.41)	(15.2, 16.3)	(1756, 4434)	(1782, 4636)	(1765, 4501)

## Data Availability

The data will be supplied if asked for.

## Supplementary Materials

The following supporting information can be downloaded at: https://www.mdpi.com/article/10.3390/ma16237349/s1. Figure S1: Lattice thermal conductivity of MgMg_2_Sb_2_ calculated using the force constant obtained at 0 K (a), 400 K (b) and 700 K (c). Figure S2: Lattice thermal conductivity of CaMg_2_Sb_2_ calculated using the force constant obtained at 0 K (a), 400 K (b) and 700 K (c). Figure S3: Non-normalized eigenvector representations for XMg_2_Sb_2_ (X=Mg or Ca) compounds at Γ point. The remaining modes are acoustic modes. Figure S4: Spectra of thermal conductivity at 700 K temperature for CaMg_2_Sb_2_ (a) and MgMg_2_Sb_2_ (b). Figure S5: Grüneisen parameters, and mean free path vs. frequency obtained from the data calculated using the 700 K ab initio MD calculated force constants. (a) Grüneisen parameters for CaMg_2_Sb_2_, (b) Grüneisen parameters for MgMg_2_Sb_2_, (c) mean free path for CaMg_2_Sb_2_, and (d) mean free path for MgMg_2_Sb_2_. Figure S6: Lifetime, and phonon group velocity vs. frequency obtained from the data calculated using the 700 K ab initio MD calculated force constants. (a) lifetime for CaMg_2_Sb_2_, (b) lifetime for MgMg_2_Sb_2_, (c) velocity for CaMg_2_Sb_2_, and (d) velocity for MgMg_2_Sb_2_. Figure S7: The MLWFs of XMg_2_Sb_2_ compound. Due to the symmetry and the similarity of the MLWFs contour for both of our considered compounds, we only shown the MLWFs around of one X (a), Mg (b) and Sb (c) atoms. Figure S8: MLWF center (WFC) distribution for XMg_2_Sb_2_ (X=Mg or Ca) compounds. The small red ball denotes the site of the WFC.
