# Supplementary material for: Lattice Thermal Conductivity in XMg2Sb2(X = Ca or Mg) Compounds: Temperature and High-Order Anharmonicity Effect
Source: Materials (Basel). 2023 Nov 25;16(23):7349. doi: 10.3390/ma16237349 (PMC10707498; doi:10.3390/ma16237349)
Supplement: Supplementary file 1 [file materials-16-07349-s001.zip › materials-2679660-supplementary.pdf]

Supplementary Materials for

Lattice Thermal Conductivity in XMg_2Sb_2(X = Ca or Mg) Compounds: Temperature and High-Order Anharmonicity Effect

Minghui Wu [1,2,*], Hongping Yang [1,2], Fengyan Xie [1,2] and Li Huang [3]

[1] College of Materials and Chemical Engineering, MinJiang University, Fuzhou 35108, China; xiefengyan@mju.edu.cn (F.X.)
[2] Fujian Key Laboratory of Functional Marine Sensing Materials, Minjiang University, Fuzhou 35108, China
[3] Department of Physics, Southern University of Science and Technology, Shenzhen 518055, China; huangl@sustech.edu.cn
* Correspondence: minghuiwu@mju.edu.cn

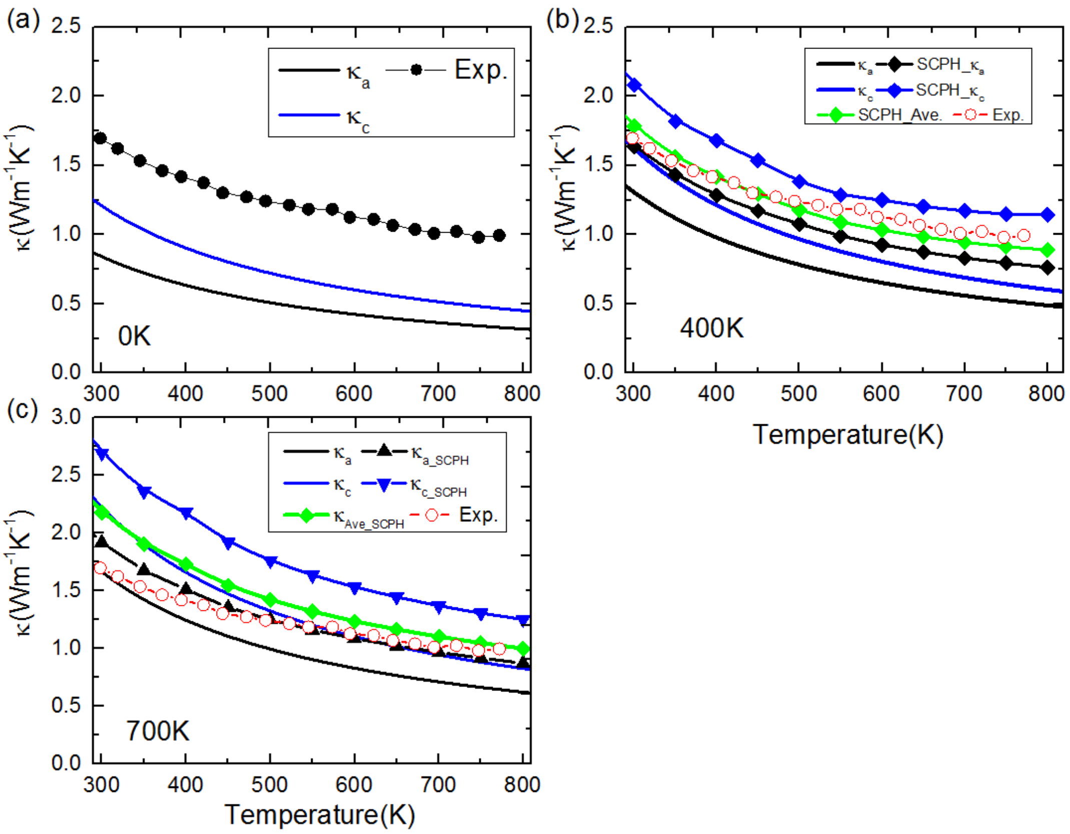

Figure S1. Lattice thermal conductivity of $MgMg_2Sb_2$ calculated using the force constant obtained at 0K (a), 400K (b) and 700K (c)

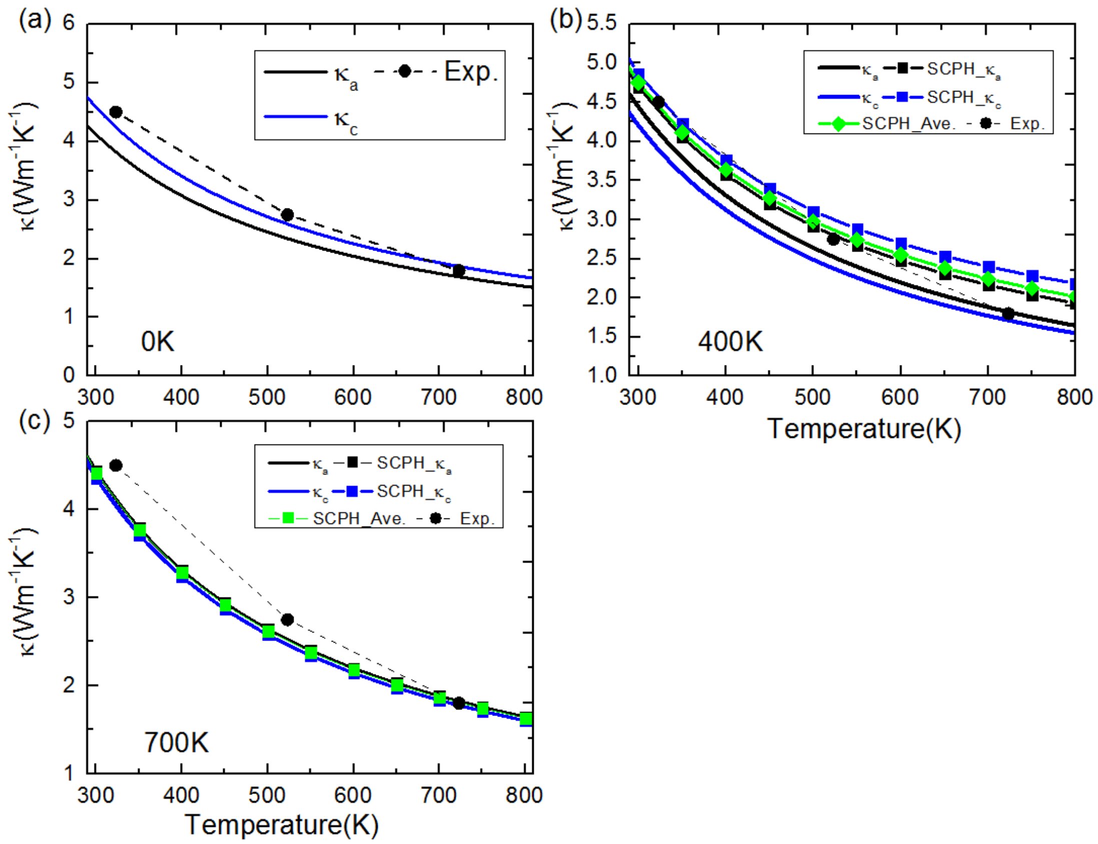

Figure S2. Lattice thermal conductivity of $CaMg_2Sb_2$ calculated using the force constant obtained at 0K (a), 400K (b) and 700K (c).

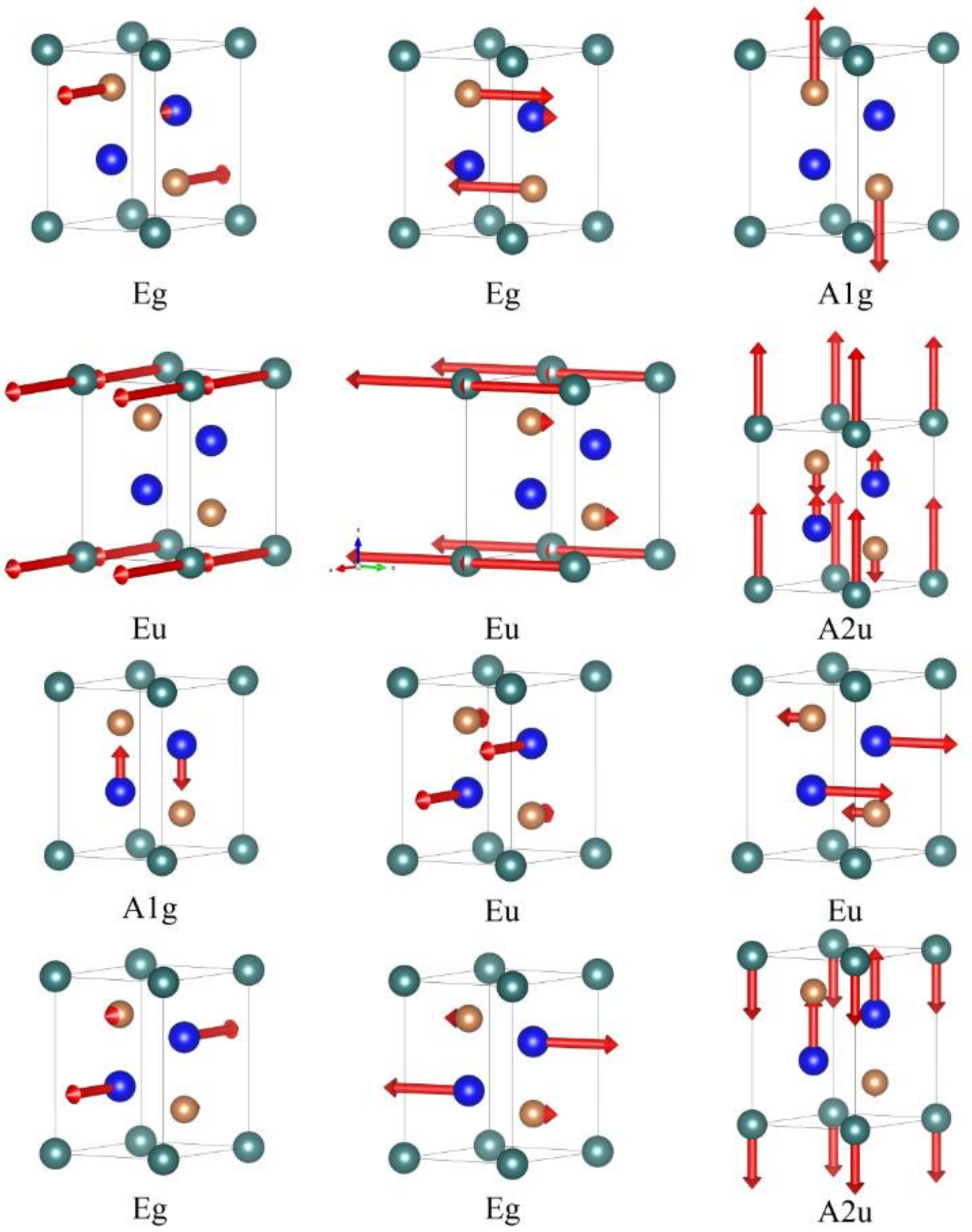

Figure S3. Non-normalized eigenvector representations for XMg_2Sb_2 (X=Mg or Ca) compounds at Γ point. The remaining modes are acoustic modes.

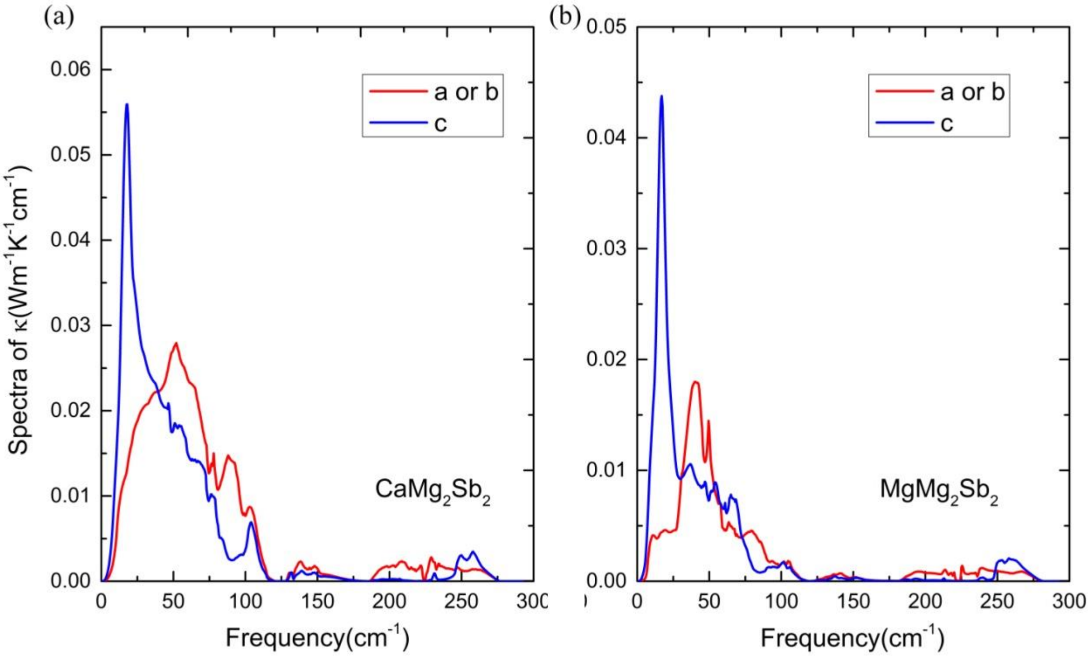

Figure S4. Spectra of thermal conductivity at 700K temperature for $CaMg_2Sb_2$ (a) and $MgMg_2Sb_2$ (b).

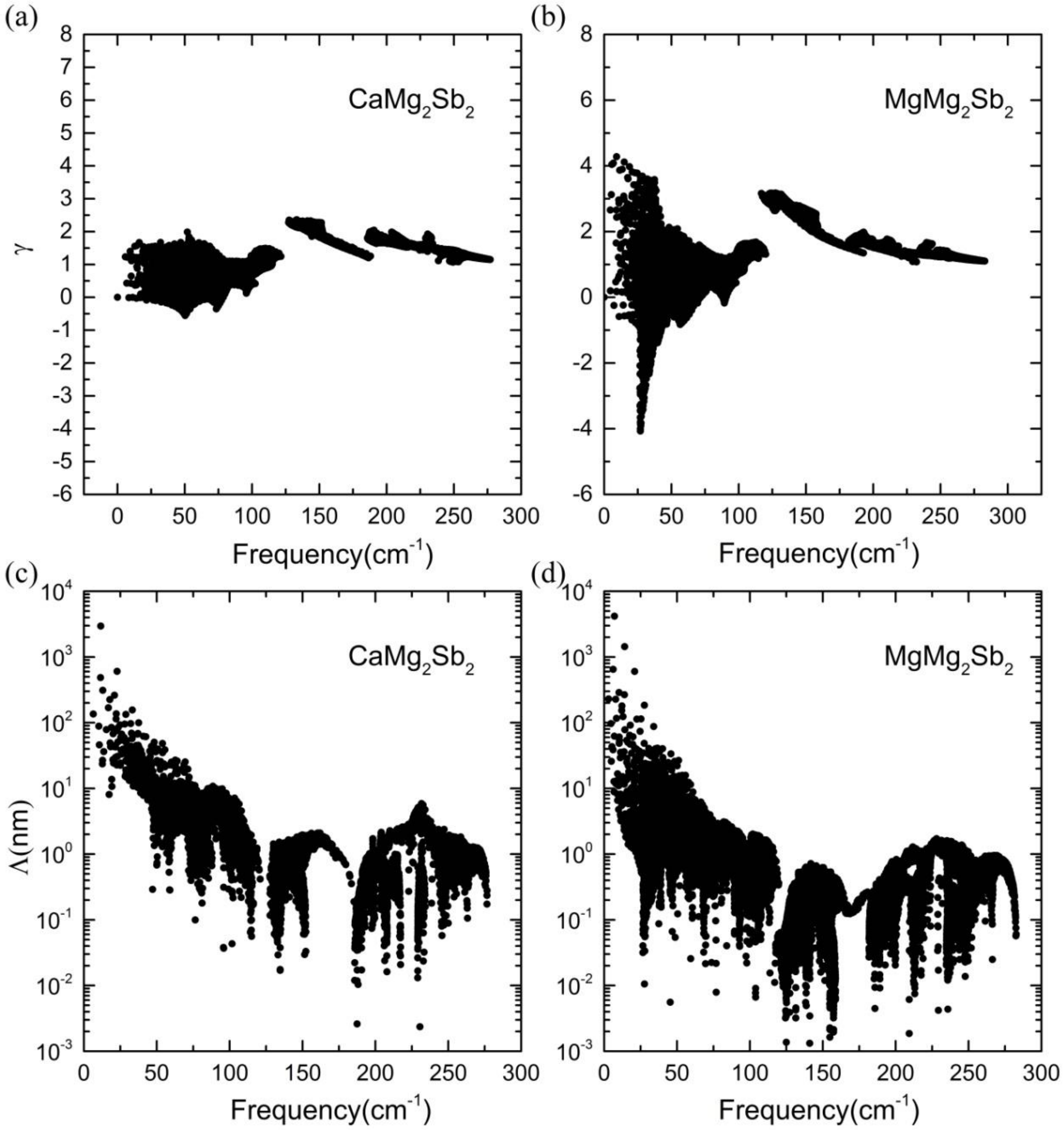

Figure S5. Grüneisen parameters, and mean free path vs. frequency obtained from the data calculated using the 700K ab initio MD calculated force constants. (a)Grüneisen parameters for $CaMg_2Sb_2$, (b) Grüneisen parameters for $MgMg_2Sb_2$, (c) mean free path for $CaMg_2Sb_2$, and (d) mean free path for $MgMg_2Sb_2$.

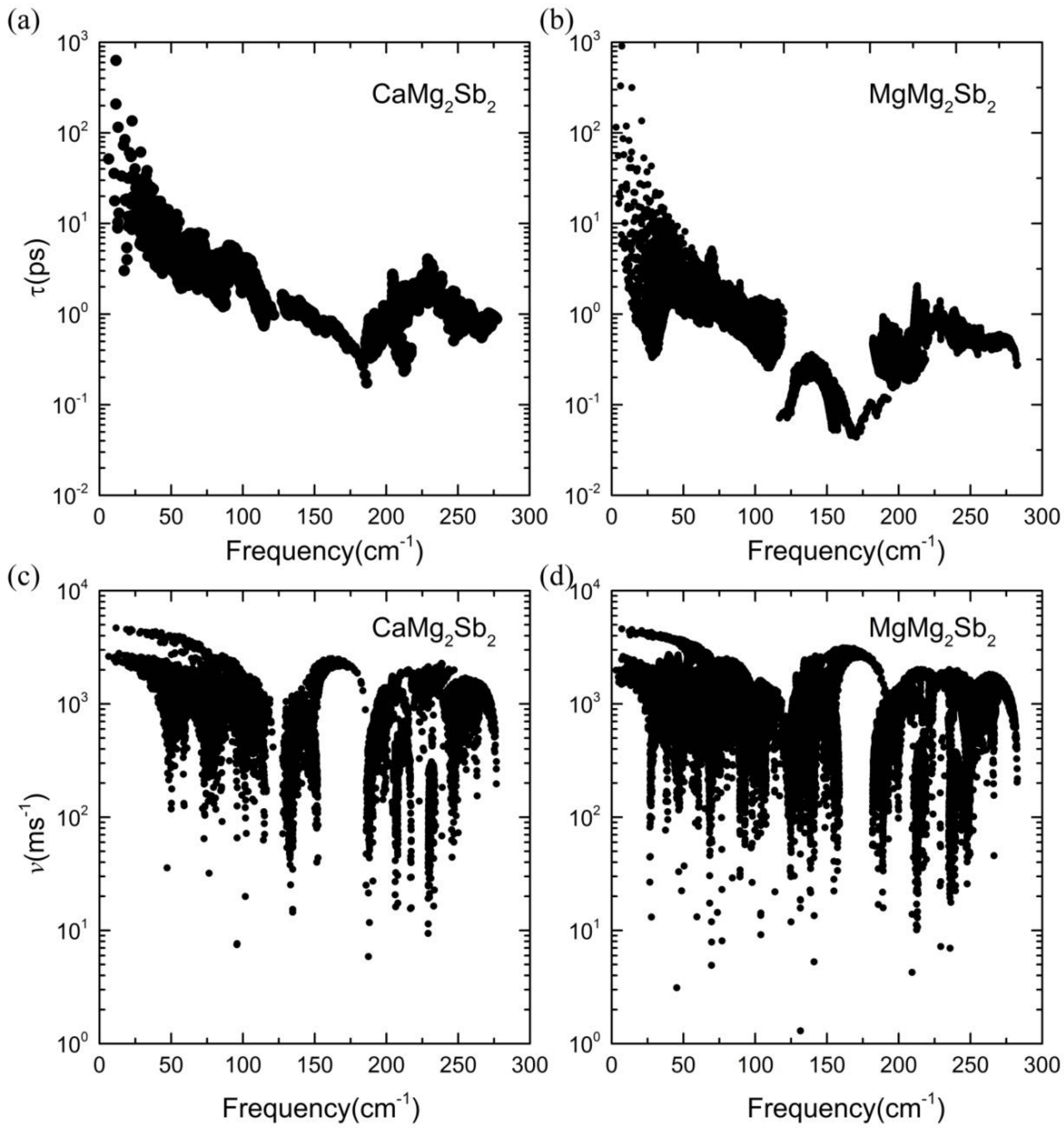

Figure S6. Lifetime, and phonon group velocity vs. frequency obtained from the data calculated using the 700K ab initio MD calculated force constants. (a) lifetime for $CaMg_2Sb_2$, (b) lifetime for $MgMg_2Sb_2$, (c) velocity for $CaMg_2Sb_2$, and (d) velocity for $MgMg_2Sb_2$.

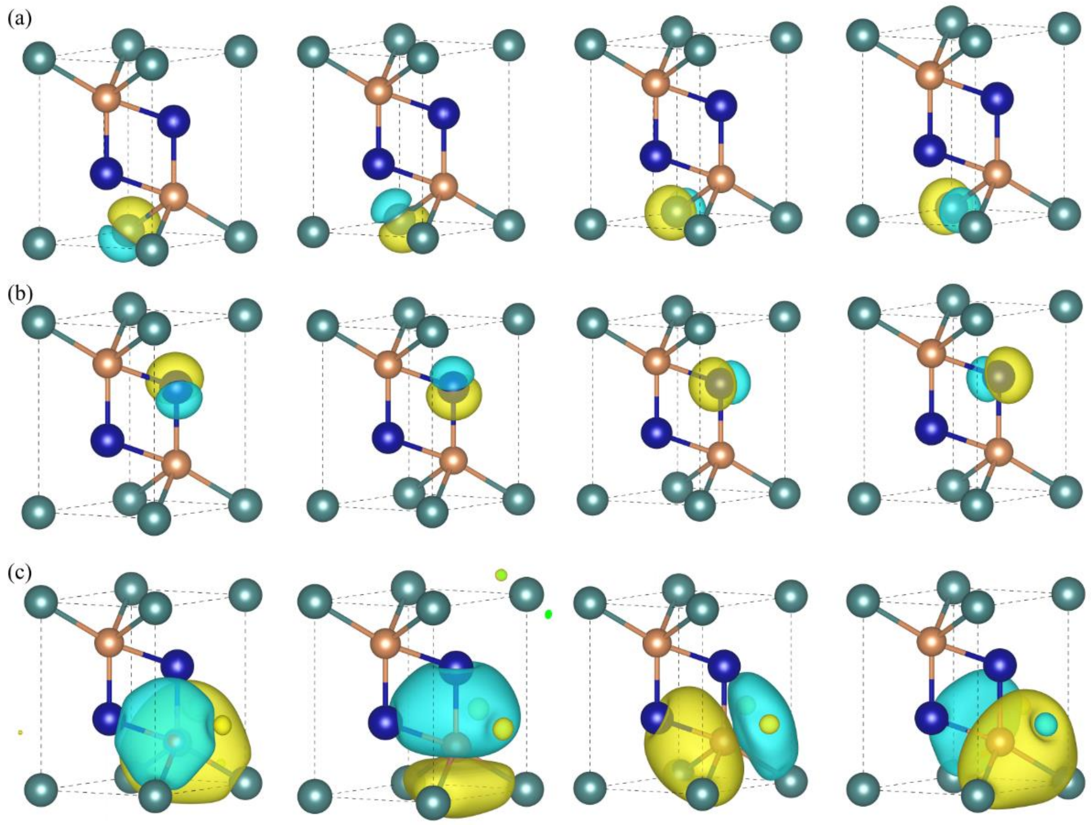

Figure S7. The MLWFs of XMg_2Sb_2 compound. Due to the symmetry and the similarity of the MLWFs contour for both of our considered compounds, we only shown the MLWFs around of one X(a), Mg (b) and Sb (c) atoms.

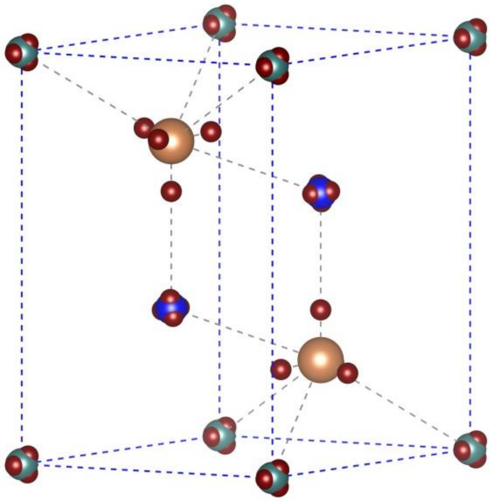

Figure S8. MLWF center (WFC) distribution for XMg_2Sb_2(X=Mg or Ca) compounds. The small red ball denotes the site of the WFC.
